# Clinical Significance and Potential Function of Complement Factor D in Acute Myeloid Leukemia

**DOI:** 10.7759/cureus.67260

**Published:** 2024-08-20

**Authors:** Taigang Zhang, Zhaozhong Li, Aoyu He, Wenjuan Zhou, Xianjin Zhu, Yanfang Song

**Affiliations:** 1 Department of Clinical Laboratory, The Affiliated People’s Hospital of Fujian University of Traditional Chinese Medicine, Fuzhou, CHN; 2 Department of Laboratory Medicine, Fujian Medical University Union Hospital, Fuzhou, CHN

**Keywords:** biomarker, prognosis, bioinformatics, aml, cfd

## Abstract

Background: Acute myeloid leukemia (AML) is a hematologic malignancy characterized by aggressive proliferation and a poor prognosis. The objective of this study is to elucidate the specific role of complement factor D (CFD) in AML, with the aim of identifying robust prognostic markers for the disease.

Methods: We performed a systematic investigation on clinical significance and potential function of CFD in AML by using the R Programming Language with The Cancer Genome Atlas (TCGA), Gene Expression Omnibus (GEO), The Human Protein Atlas (HPA), The University of ALabama at Birmingham CANcer data analysis Portal (UALCAN), Gene Expression Profiling Interactive Analysis (GEPIA), Kaplan-Meier plotter, Cancer Cell Line Encyclopedia (CCLE) database, and Comprehensive Analysis on Multi-Omics of Immunotherapy in Pan-cancer (CAMOIP) database. The expression of CFD in AML patients was verified by reverse transcription-quantitative polymerase chain reaction (RT-qPCR).

Results: The expression of CFD was the highest in AML cells than in other tumor cell lines. The expression of CFD was also higher in AML patients than in the matched normal group. Compared with the low expression of the CFD group, high expression of CFD predicted better overall survival (OS) and lower tumor mutational burden (TMB) in AML patients. Moreover, a nomogram model based on CFD was successfully constructed to predict the OS of AML patients. Notably, the expression of CFD was associated with drug sensitivity and monocyte cell infiltration.

Conclusion: CFD could serve as a potential OS prognostic biomarker and guide clinical treatment for AML.

## Introduction

Acute myeloid leukemia (AML) is one of the most common hematological malignancies, with 20,800 (1.0%) estimated new cases and 11,220 (1.8%) deaths in 2024 worldwide [1,2]. Despite advancements in combination chemotherapy that have improved patient survival rates, achieving a complete cure for AML remains challenging, with many patients experiencing relapses and developing resistance to chemotherapy [3,4]. Consequently, identifying robust prognostic markers is crucial for optimizing AML treatment.

Complement factor D (CFD), an important component of the alternative complement pathway [5,6], plays a crucial role in regulating complement activation [7]. More and more studies show that abnormal expression of CFD can lead to impairing complement activation homeostasis, inducing inflammation, and promoting malignant cell proliferation, migration, and invasion [8-11]. Increased CFD is associated with worse progression-free survival in colorectal cancer [12] and promotes breast cancer growth and cancer stem cell-like properties [11], and CFD inhibitor is considered a highly sensitive and specific drug candidate in the therapy of cutaneous squamous cell carcinoma [13]. These studies suggest a significant role for CFD in tumorigenesis. However, the role of CFD in AML has not yet been elucidated.

In this study, we analyzed CFD expression in AML cell lines and patient samples and explored its prognostic significance in AML. Additionally, we investigated the association of CFD with genetic variants, immune infiltration, and drug sensitivity in AML patients. Our findings, although requiring further validation, provide novel insights into the prognostic roles of CFD in AML.

## Materials and methods

Data sourcing

To compare the differences in CFD expression between normal subjects and AML patients, we analyzed and downloaded the differential gene expression data of GSE9476 (https://www.ncbi.nlm.nih.gov/geo/query/acc.cgi?acc=GSE9476) by GEO2R. Also, to analyze the relationship between CFD and the prognosis of AML patients, we downloaded transcriptomic data and corresponding clinical information of 151 AML patients from The Cancer Genome Atlas (TCGA) database. Further to explore the expression of CFD in cell lines, we downloaded CFD expression data from the Cancer Cell Line Encyclopedia (CCLE) database (https://sites.broadinstitute.org/ccle/) for cells derived from different tissues [14].

The expression of CFD in AML cell lines and tissues

We first analyzed CFD mRNA expression in AML cell lines using RNA sequencing (RNA-seq) data from the Human Protein Atlas (HPA) (https://www.proteinatlas.org/) [15] and the CCLE databases. The HPA database provides transcriptomic and proteomic data from normal and pathological human tissues through RNA-seq and immunohistochemistry (IHC). CFD expression was evaluated across various cell lines, including leukemia, lung, breast, and brain cell lines. The CCLE project offers comprehensive genetic characterization, including RNA-seq data, for over 1,100 human cancer cell lines from diverse lineages and ethnicities, with a specific focus on AML cell lines. Data from CCLE were visualized using the R package "ggplot2" (The R Foundation for Statistical Computing, Vienna, Austria).

Next, we investigated CFD expression in AML patients using several databases, including the University of ALabama at Birmingham CANcer data analysis Portal (UALCAN) (http://ualcan.path.uab.edu/), Gene Expression Profiling Interactive Analysis (GEPIA) (http://gepia.cancer-pku.cn), and the GSE9476 dataset [16-18]. GEPIA, which includes data from 9,736 tumor samples and 8,587 normal tissue samples from the TCGA and GTEx projects, was used for single-gene analysis to generate plots of CFD expression in AML and normal tissues using default thresholds. The GSE9476 dataset from the Gene Expression Omnibus (GEO) database provided CFD expression profiles in normal hematopoietic cells from 38 healthy donors and leukemic blasts from 26 AML patients.

Expression validation by RT-qPCR

This study was performed in line with the principles of the Declaration of Helsinki, and this study was approved by the Ethics Committee of the Fujian Medical University Union Hospital. After informed consent was obtained from all individual participants enrolled in the study, we obtained venous whole blood from 12 AML patients and 12 healthy individuals at Fujian Medical University Union Hospital, and peripheral blood mononuclear cells (PBMC) were enriched by density gradient centrifugation. Total RNA was extracted from PBMC using the RNA extraction kit (centrifugal column method) (Beyotime Biotechnology, Jiangsu, China) following the manufacturer's instructions. Total RNA was reverse transcribed into first-strand cDNA using the RevertAidTM First Strand cDNA Synthesis Kit (Thermo Scientific, Maryland, USA). Real-time polymerase chain reaction (PCR) was conducted by using the SYBR Green Master Mix Kit (Applied Biosystems, Foster City, USA). The primers used in this study are shown in Table 1.

**Table 1:** Sequences of primers used in PCR PCR: polymerase chain reaction

Gene	Primer Type	Primers Sequence (5’—3’)
CFD	Forward	GGGTCACCCAAGCAACAAAG
	Reverse	CGTGGCCCATGCTGATCTC
GAPDH	Forward	CACATGGCCTCCAAGGAGTAA
	Reverse	TGAGGGTCTCTCTCTTCCTCTTGT

Survival analysis of CFD in AML

Overall survival (OS) and event-free survival (EFS) in AML patients were analyzed by the Kaplan-Meier method. We used the R package “Survival” to analyze the data from TCGA, and the Kaplan-Meier Plotter (https://kmplot.com/analysis/) [19] was used to analyze the data from GSE1159 and GSE6891. The GSE1159 dataset included a total of 285 AML patients and eight healthy control subjects. The GSE6891 dataset included 537 AML patients. We all used the median CFD expression as a threshold to divide into a CFD high expression group and a CFD low expression group.

Independent prognostic analysis

We removed five patients who were not treated and two patients whose cytogenetics were not reported of 151 AML patients from the TCGA database. Univariate and multi-factorial Cox regression analyses were performed to determine whether CFD expression as a single factor could predict the prognosis of AML patients in combination with other clinical characteristics. We selected the factors (p<0.05) in the univariate Cox regression analysis for the multifactorial Cox regression analysis.

Nomogram established based on CFD expression and clinical features

The prognosis-related nomogram survival model established by the R package “Regplot” was applied to assess the probability of survival at one, three, and five years in patients with AML. On the basis of the bootstrap resampling method, we evaluated the predictive function of the nomogram by the calibration curve.

Immune infiltration analysis and genetic mutation analysis

The analysis was based on the online database Comprehensive Analysis on Multi-Omics of Immunotherapy in Pan-cancer (CAMOIP) (http://220.189.241.246:13838/) [20]. The relationship between CFD expression and AML mutational frequencies and tumor mutation burden (TMB) was analyzed. At the same time, “CIBERSORT” and “quanTIseq” algorithms were used to analyze the correlation between CFD expression and immune cell infiltration.

Drug sensitivity assessment by pRRophetic

We utilized the "pRRophetic" R package to investigate the variances in drug sensitivity between the two subcategories. The accuracy of drug sensitivity was projected by analyzing the half-maximal inhibitory concentration (IC50).

## Results

CFD was highly expressed in AML

**Figure 1:**
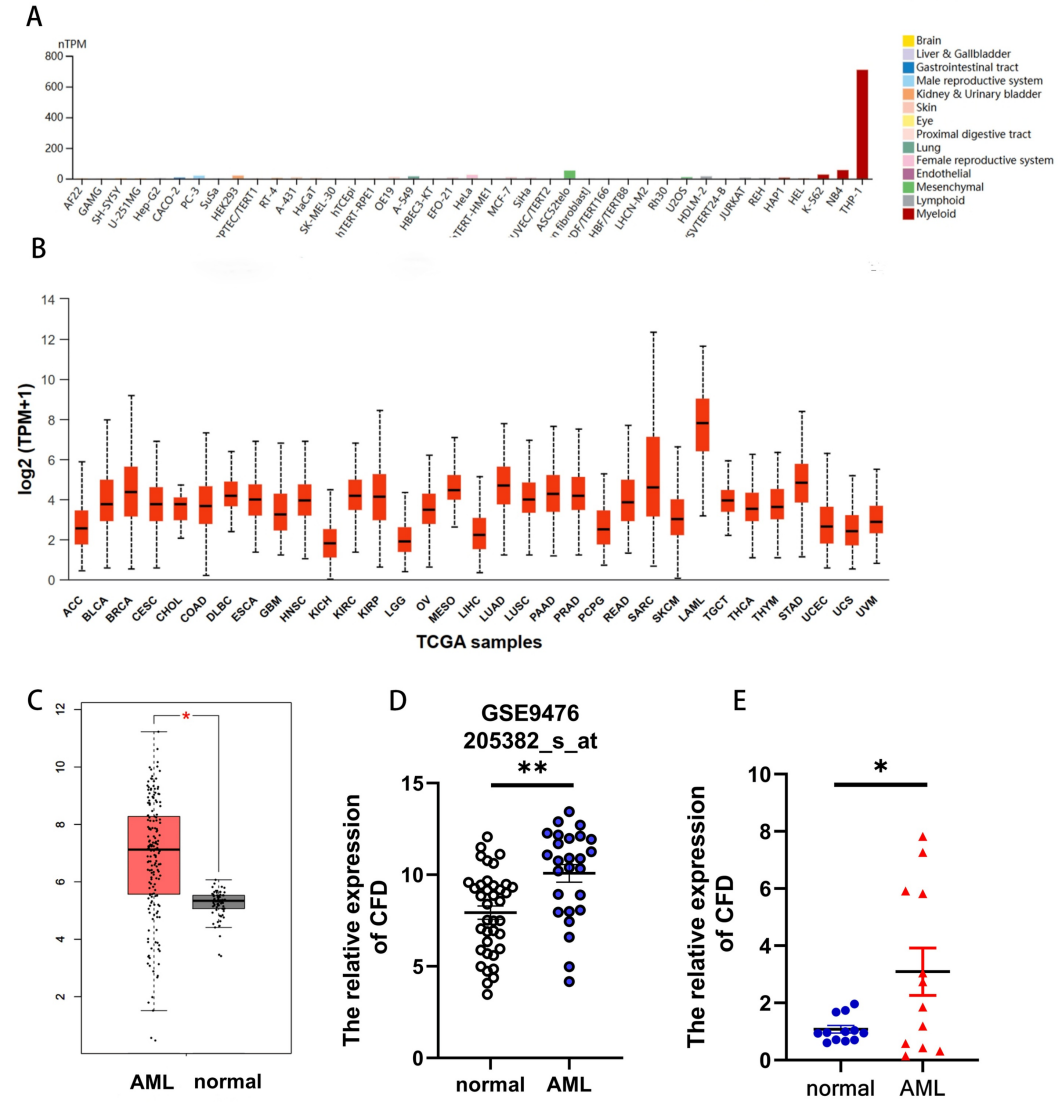
The expression of CFD was increased in AML. (A) The expression of complement factor D (CFD) in cancer cell lines (HPA). (B) The expression of CFD in pan-cancer from UALCAN. (C) The expression of CFD in acute myeloid leukemia (AML) and normal tissues from GEPIA. (D) The expression of CFD in AML and normal from the GEO database (GSE9476). (E) PBMC from 12 AML patients and 12 healthy individuals were collected and the expression of CFD was analyzed using RT-qPCR. *p<0.05; **p<0.01. HPA: Human Protein Atlas; UALCAN: University of ALabama at Birmingham CANcer data analysis Portal; GEPIA: Gene Expression Profiling Interactive Analysis; GEO: Gene Expression Omnibus; PBMC: peripheral blood mononuclear cells; RT-qPCR: reverse transcription-quantitative polymerase chain reaction; ACC: adrenocortical carcinoma; BLCA: urothelial bladder carcinoma; BRCA: breast invasive carcinoma; CESC: endocervical adenocarcinoma; CHOL: cholangiocarcinoma; COAD: colon adenocarcinoma; DLBC: diffuse large B-cell lymphoma; ESCA: esophageal carcinoma; GBM: glioblastoma multiforme; HNSC: head and neck squamous cell carcinoma; KICH: kidney chromophobe; KIRC: kidney renal clear cell carcinoma; KIRP: kidney renal papillary cell carcinoma; LGG: brain lower grade glioma; OV: ovarian serous cystadenocarcinoma; MESO: mesothelioma; LIHC: liver hepatocellular carcinoma; LUAD: lung adenocarcinoma; LUSC: lung squamous cell carcinoma; PAAD: pancreatic adenocarcinoma; PRAD: prostate adenocarcinoma; PCPG: pheochromocytoma and paraganglioma; READ: rectum adenocarcinoma; SARC: sarcoma; SKCM: skin cutaneous melanoma; LAML: acute myeloid leukemia; TGCT: testicular germ cell tumors; THCA: thyroid carcinoma; THYM: Thymoma; STAD: stomach adenocarcinoma; UCEC: uterine corpus endometrial carcinoma; UCS: uterine carcinosarcoma; UVM: uveal melanoma

To demonstrate the role of CFD in AML, we first analyzed the expression of CFD in AML cell lines. The results from the HPA database showed that the expression of CFD was highest in AML cell lines compared with other tumor cell lines (Figure 1A). Results from the CCLE database showed the same result (see Figure 7 in Appendices). Next, we analyzed the expression of CFD in AML patients using the UALCAN database and found that the expression of CFD was highest in AML patients in comparison with other tumor patients (Figure 1B). In addition, compared with the normal group, the expression of CFD was increased in AML patients using the GEPIA database (Figure 1C) and the GEO dataset GSE9476 (Figure 1D). Importantly, we confirmed that the expression of CFD was increased in PBMC from patients with AML (p<0.05) using RT-qPCR (Figure 1E). Taken together, these data showed that the expression of CFD was increased in AML.

CFD expression was associated with the prognosis of AML patients

**Figure 2:**
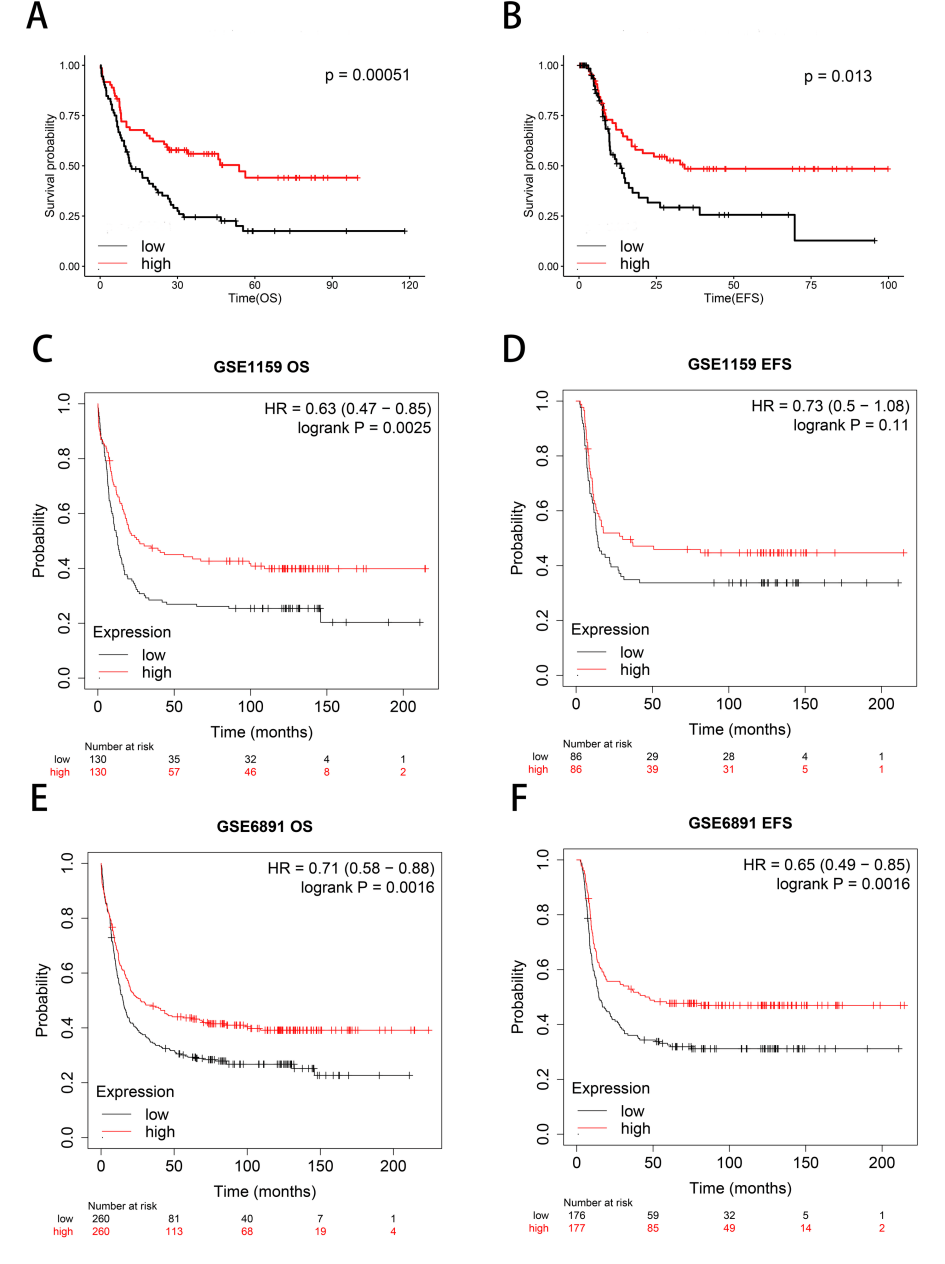
CFD survival analysis for AML (A) Analysis of CFD with OS in AML from the TCGA cohort. (B) Analysis of CFD with EFS in AML from the TCGA cohort. (C) Analysis of CFD with OS in AML from the GSE1159 dataset. (D) Analysis of CFD with EFS in AML from the GSE1159 dataset. (E) Analysis of CFD with OS in AML from the GSE6891 dataset. (F) Analysis of CFD with EFS in AML from the GSE6891 dataset. CFD: complement factor D; AML: acute myeloid leukemia; TCGA: The Cancer Genome Atlas; OS: overall survival; EFS: event-free survival

To explore the role of CFD in AML prognosis, the TCGA data was analyzed and the results showed that high expression of CFD was associated with excellent OS (p=0.00051) and EFS (p=0.013) in AML patients (Figures 2A, 2B). In addition, the results of the GSE1159 dataset also showed that patients with high expression of CFD had higher OS (p=0.0025) in AML (Figure 2C), and we also found that CFD overexpression was related to higher EFS (Figure 2D), albeit statistical significance was not achieved (p=0.11). A similar investigation was conducted on the GSE6891 dataset, which demonstrated that the overexpression of CFD was associated with both favorable OS (p=0.0016) and EFS (p=0.0016) (Figures 2E, 2F). These results suggested that the expression of CFD has a close relation with OS and EFS of AML, which implies the patient with high expression of CFD has a better prognosis.

CFD could be an independent factor in predicting the prognosis of AML patients

To further explore the role of CFD in AML prognosis, we performed univariate COX regression analysis as well as multivariate COX regression analysis to determine whether CFD expression, as an independent factor, could predict the OS and EFS of AML patients. The factors with p<0.05 in the one-way Cox regression analysis were selected for the multifactor Cox regression analysis. The one-way Cox regression analysis revealed a significant association between low CFD expression and OS (hazard ratio (HR)=2.09, 95% confidence interval (CI): 1.37-3.2, p=0.001), indicating that as CFD expression levels decrease, OS outcomes deteriorate. Additionally, low CFD expression was found to be significantly associated with OS in a multifactorial Cox regression analysis (HR=2.5, 95% CI: 1.29-4.84, p=0.0066) (Table 2). The results of the one-way COX analysis indicated that low CFD expression was significantly associated with EFS (HR=1.84, 95% CI: 1.13-2.99, p=0.014). The multifactorial COX analysis yielded no significant association between low CFD expression and EFS (HR=1.5, 95% CI: 0.8-2.82, p=0.2057) (Table 3). The results suggested that CFD could be an independent prognostic factor to predict the OS of AML patients. Unfortunately, CFD expression could not be used as an independent prognostic factor for EFS in AML patients.

**Table 2:** Correlations between CFD for OS and clinical characteristics by univariate Cox regression analysis and multivariate Cox regression analysis N: number of patients; FAB: French–American–British subtypes; BM BLAST: bone marrow blast; PB BLAST: peripheral blood blast; WBC: white blood cell; CFD: complement factor D; OS: overall survival; HSCT: hematopoietic stem cell transplantation

	Univariate analysis		Multivariate analysis	
Variants	Hazard ratio (95% CI)	p-value	Hazard ratio (95% CI)	p-value
Age				
<65	0.3 (0.2-0.46)	<0.001	0.55 (0.32-0.93)	0.0262
BM BLAST	1 (0.99-1.01)	0.706	-	-
CFD expression				
Low	2.09 (1.37-3.2)	0.001	2.5 (1.29-4.84)	0.0066
Cytogenetic				
Complex	0.92 (0.34-2.45)	0.862	-	-
(7q)/7q-	1.16 (0.35-3.79)	0.811	-	-
inv(16)	0.32 (0.08-1.29)	0.11	-	-
Normal	0.81 (0.34-1.89)	0.619	-	-
others	0.79 (0.22-2.81)	0.713	-	-
t(15;17)	0.37 (0.1-1.32)	0.126	-	-
t(8;21)	0.39 (0.1-1.57)	0.185	-	-
FAB				
M1	0.96 (0.46-2.02)	0.915	2 (0.92-4.36)	0.0808
M2	0.84 (0.4-1.77)	0.643	1.28 (0.59-2.78)	0.5246
M3	0.28 (0.09-0.9)	0.033	0.82 (0.15-4.35)	0.8142
M4	1 (0.47-2.13)	0.991	1.82 (0.8-4.12)	0.1535
M5	1.1 (0.45-2.72)	0.829	2 (0.69-5.82)	0.203
M6	2.7 (0.58-12.48)	0.204	2.55 (0.55-11.85)	0.2324
M7	2.14 (0.27-16.87)	0.471	1.05 (0.13-8.56)	0.9621
PB BLAST	1 (0.99-1.01)	0.661	-	-
Risk cytogenetic				
Intermediate	2.89 (1.47-5.68)	0.002	2.24 (1.01-4.97)	0.0461
Poor	3.99 (1.91-8.34)	<0.001	3.68 (1.47-9.19)	0.0053
Sex				
Male	1 (0.66-1.51)	0.987	-	-
Therapy				
HSCT	0.56 (0.37-0.86)	0.007	0.34 (0.19-0.61)	3.00E-04
WBC/10^9^/L	1 (1-1.01)	0.086	-	-

**Table 3:** Correlations between CFD for EFS and clinical characteristics by univariate Cox regression analysis and multivariate Cox regression analysis N: number of patients; FAB: French–American–British subtypes; BM BLAST: bone marrow blast; PB BLAST: peripheral blood blast; WBC: white blood cell; CFD: complement factor D; EFS: event-free survival; HSCT: hematopoietic stem cell transplantation

	Univariate analysis		Multivariate analysis	
Variants	Hazard ratio (95% CI)	p-value	Hazard ratio (95% CI)	p-value
Age				
<65	0.64 (0.37-1.13)	0.127	-	-
BM BLAST	1 (0.98-1.01)	0.622	-	-
CFD expression				
Low	1.84 (1.13-2.99)	0.014	1.5 (0.8-2.82)	0.2057
Cytogenetic				
Complex	1.78 (0.37-8.57)	0.473	-	-
(7q)/7q-	1.24 (0.17-8.83)	0.828	-	-
inv(16)	1.78 (0.34-9.18)	0.492	-	-
Normal	2.16 (0.52-8.94)	0.288	-	-
others	1.04 (0.14-7.39)	0.972	-	-
t(15;17)	0.53 (0.07-3.79)	0.528	-	-
t(8;21)	0.92 (0.15-5.54)	0.932	-	-
FAB				
M1	1.05 (0.44-2.54)	0.908	0.87 (0.32-2.35)	0.7782
M2	0.86 (0.35-2.09)	0.735	0.83 (0.31-2.19)	0.708
M3	0.18 (0.04-0.87)	0.033	0.4 (0.07-2.47)	0.3236
M4	1.05 (0.43-2.58)	0.913	1.24 (0.44-3.52)	0.6869
M5	0.91 (0.31-2.72)	0.866	1.21 (0.3-4.9)	0.7857
M6	9.39 (1.84-47.87)	0.007	19.13 (3.44-106.3)	0.0007
M7	4.96 (0.59-41.82)	0.141	5.49 (0.6-50.04)	0.1313
PB BLAST	1.01 (1-1.02)	0.016	1.01 (1-1.02)	0.1097
Risk cytogenetic				
Intermediate	2.73 (1.38-5.43)	0.004	1.67 (0.75-3.72)	0.2085
Poor	1.66 (0.67-4.09)	0.273	0.82 (0.27-2.48)	0.7204
Sex				
Male	0.94 (0.58-1.52)	0.805	-	-
Therapy				
HSCT	1.54 (0.94-2.54)	0.087	-	-
WBC/10^9^/L	1.01 (1-1.01)	0.003	1 (1-1.01)	0.4236

Successfully establishing a nomogram for predicting OS of AML patients

**Figure 3:**
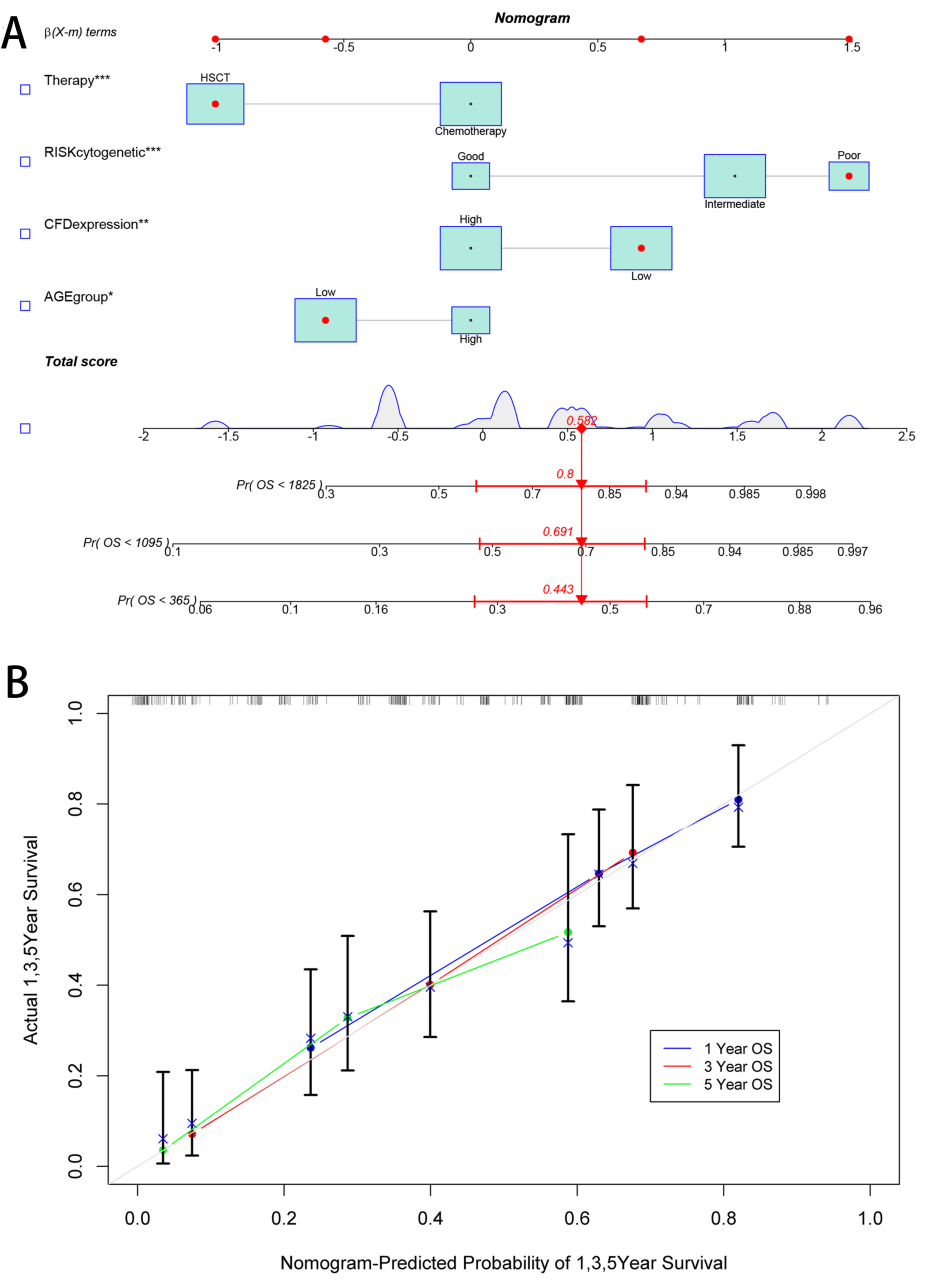
Construction of AML nomogram model (A) Nomogram for predicting one-, three-, or five-year OS; (B) calibration plots for predicting one-, three-, or five-year OS. *p<0.05; **p<0.01; ***p<0.001. AML: acute myeloid leukemia; OS: overall survival

Next, we employed the "regplot" package in R to create a nomogram model of “CFD expression”, “Therapy”, “Age group”, and “RISK cytogenetic” to predict the OS of AML patients. The nomogram model could predict one-, three-, and five-year survival in AML patients (Figure 3A). The calibration curve indicated that the projected values corresponded reasonably well with the real patient survival rate (Figure 3B), suggesting that we successfully established a nomogram for predicting the OS of AML patients. The model could accurately predict one-, three-, and five-year survival of AML patients.

The expression of CFD was associated with mutational frequencies and tumor mutational burden (TMB)

**Figure 4:**
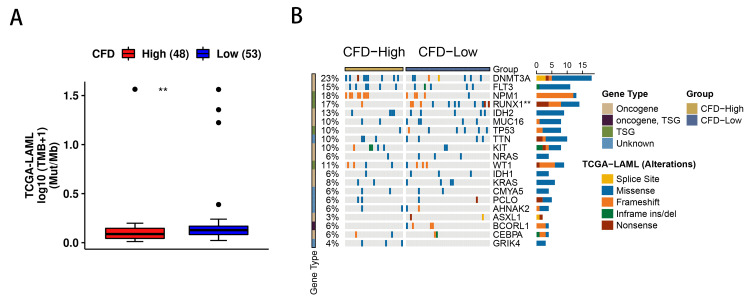
The relationship between CFD and AML mutation (A) Comparison of TMB in the CFD high expression group and low expression group in AML. (B) Gene mutational frequencies in the CFD high expression group and low expression group in AML. *p<0.05; **p<0.01. CFD: complement factor D; AML: acute myeloid leukemia; TMB: tumor mutational burden

It is well known that the occurrence of AML is closely related to genetic mutations [21]. To investigate whether CFD was associated with mutations in AML genes, we analyzed the TMB between high and low CFD expression groups using the CAMOIP database and mapped the mutation landscape. The results showed that there was a significant difference in the TMB index of AML between the high and low CFD expression groups (Figure 4A), meanwhile, in the low-expression group of CFD, there were more mutations in Runt-related transcription factor 1 (RUNX1), indicated that CFD may be associated with the mutation of RUNX1 (Figure 4B).

The expression of CFD was associated with monocyte cell infiltration of AML patients

**Figure 5:**
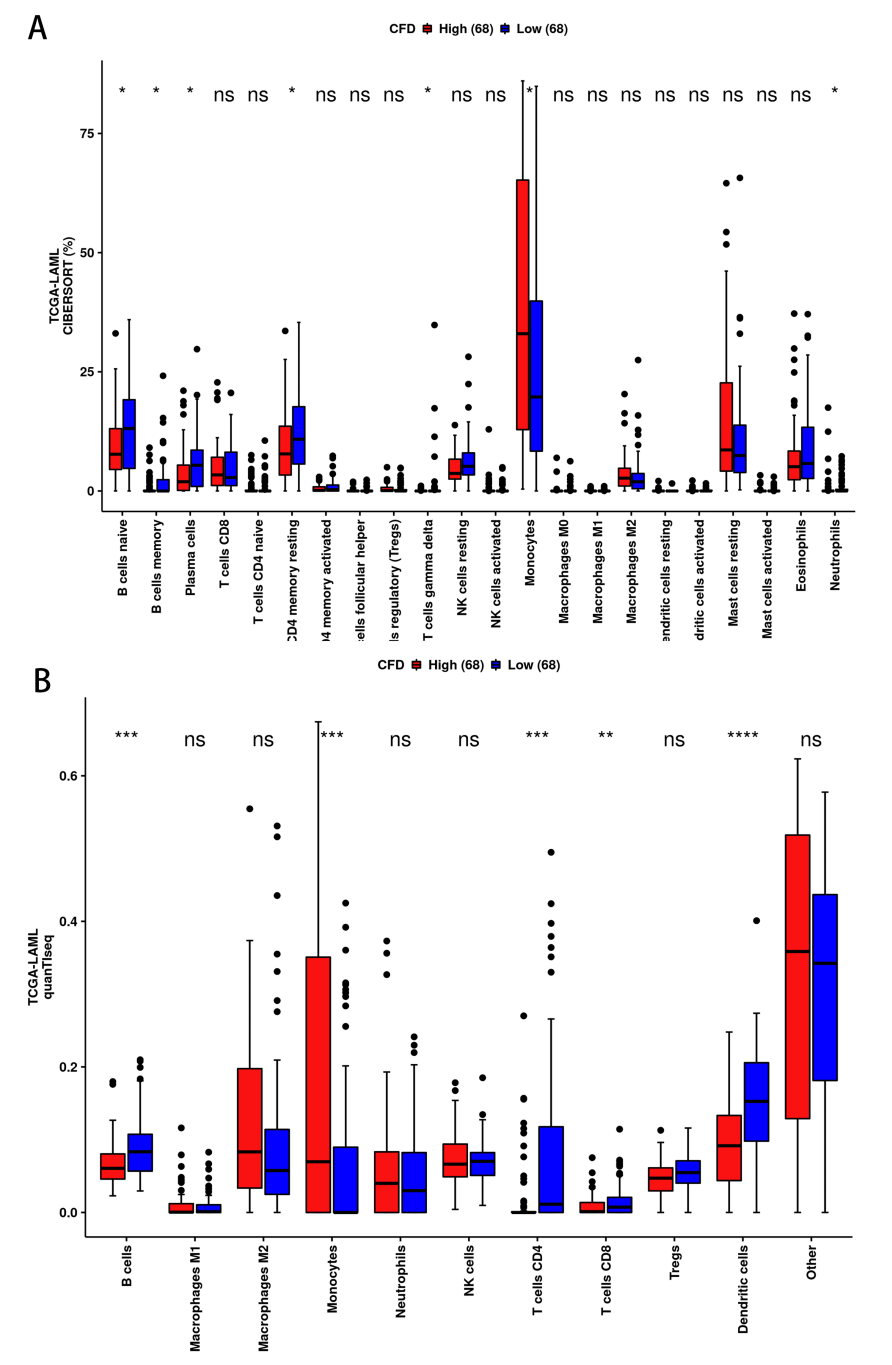
Immune cell infiltration analysis of AML (A) Comparison of immune cell infiltration in the CFD high expression group and low expression group by CIBERSORT. (B) Comparison of immune cell infiltration in the CFD high expression group and low expression group by quanTIseq.*p<0.05; **p<0.01; ***p<0.001. AML: acute myeloid leukemia; CFD: complement factor D

A growing number of studies have confirmed that immune cell infiltration is involved in cancer onset, progression, metastasis, and immune escape and is associated with prognosis [22]. In order to investigate the potential involvement of CFD in the immune processes of AML, we utilized the CAMOIP database to categorize the TCGA-LAML data into two groups based on high and low CFD expression levels. Subsequently, we employed immunological analysis using various algorithms including CIBERSORT, quanTIseq. We also divided AML patients into high CFD expression and low CFD expression groups, and we found that monocytes infiltrated more in the high CFD expression group in both algorithms' results (p<0.05 for both) (Figure 5).

The expression of CFD was associated with drug sensitivity of AML patients

**Figure 6:**
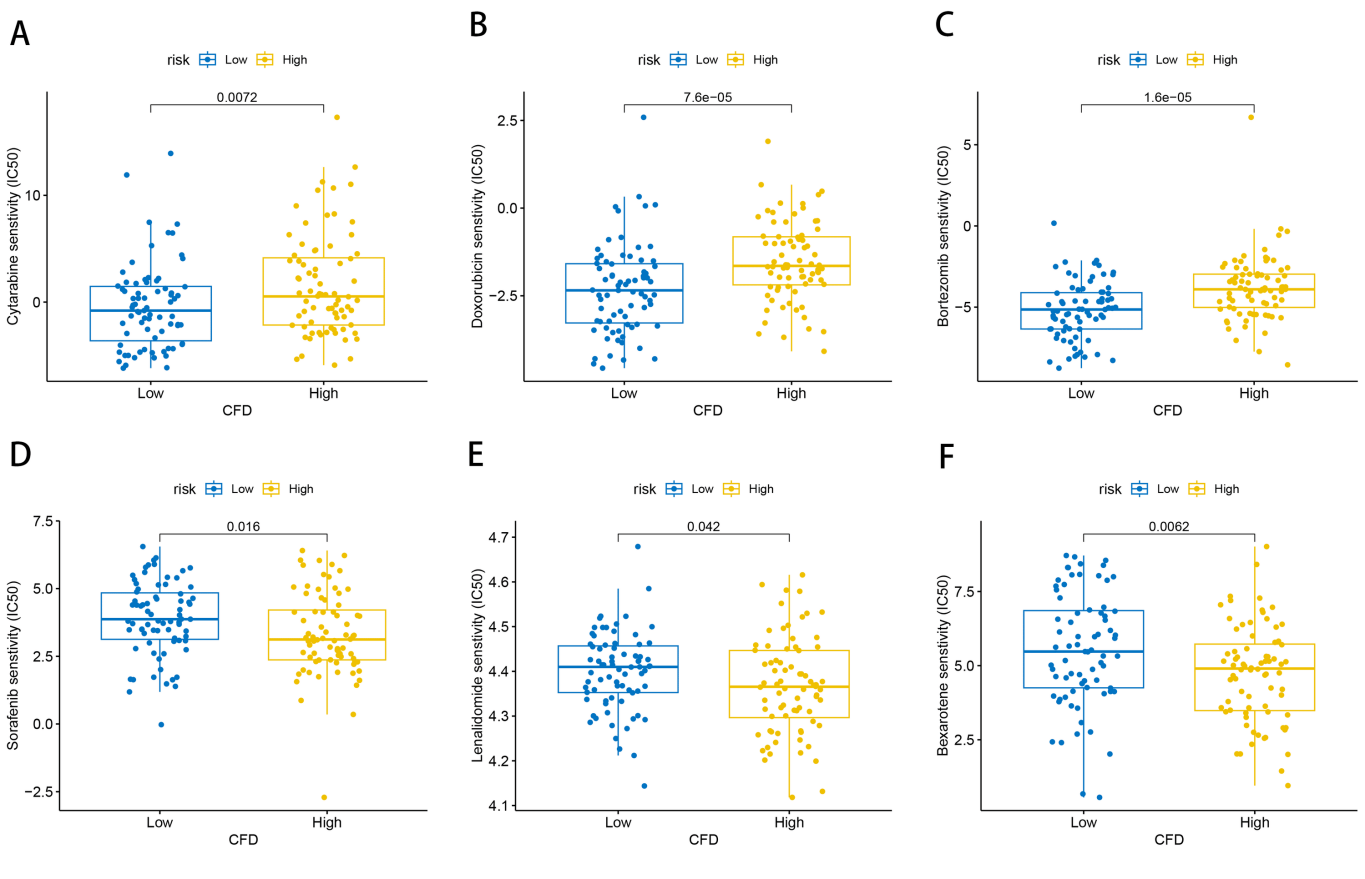
Drug sensitivity analysis in the CFD high expression group and low expression group All p-values were less than 0.05. CFD: complement factor D

**Figure 7:**
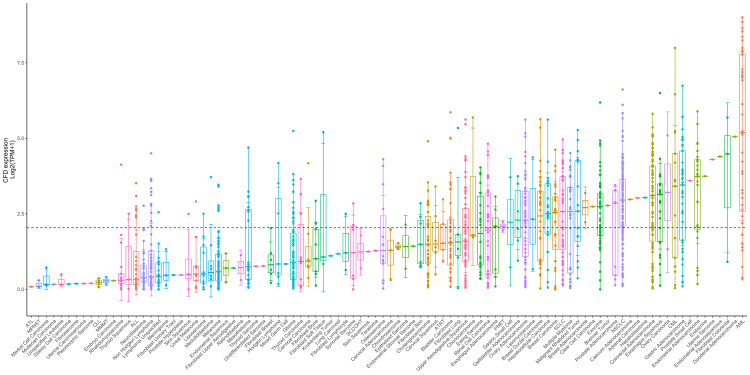
The expression of CFD in cancer cell lines (CCLE) CFD: complement factor D

To explore the role of CFD in guiding treatment, we used the R package “pRRophetic” to analyze the relationship between the expression of CFD and drug sensitivity, and the findings indicated that patients with low CFD expression showed decreased IC50 values for cytarabine, doxorubicin, and bortezomib (p<0.01 for all) (Figures 6A-6C). It is suggested that individuals in the low CFD expression group may demonstrate heightened sensitivity to these drugs. In contrast, IC50 values for sorafenib, lenalidomide, and bexarotene were found to be lower in patients with high CFD expression (p<0.05 for all) (Figures 6D-6F). Thus, sorafenib, lenalidomide, and bexarotene might offer enhanced efficacy in patients with high CFD expression levels.

## Discussion

Chemotherapy is currently the most effective treatment for AML, but more than 30% of patients relapse or develop drug resistance [23]. Therefore, it is essential to find a robust prognostic marker to provide optimal treatment. In the present study, we found that the expression of CFD was increased in AML, and high expression of CFD was associated with a good OS of AML patients. We also found that the expression of CFD was associated with TMB, drug sensitivity, and monocyte cell infiltration. Our study suggests that CFD can be used as a potential prognostic biomarker for AML.

CFD, also known as adipsin, is an important component of the alternative complement pathway. Although the role of CFD in tumors has been demonstrated, such as cutaneous squamous cell carcinoma [13], colorectal cancer [12], and breast cancer [11]; the role of CFD in AML has not been widely demonstrated. Our study found increased CFD expression in AML, and notably, high CFD expression was an independent factor for OS, with patients exhibiting high CFD levels showing excellent OS. Previous studies have found that high expression of CFD is associated with poor prognosis in adrenocortical carcinoma, thyroid cancer, uveal melanoma, low-grade glioma, and glioblastoma [24]. This inconsistency indicates that CFD may play different roles in different tumors. Our findings, for the first time, highlight the prognostic role of CFD in hematological tumors. In addition, our study also showed that AML patients with high expression of CFD have low TMB and RUNX1 mutational frequencies. Previous studies have indicated that AML patients with high TMB and RUNX1 mutational frequencies have poorer OS [25-27]. Given the key role of OS rates, TMB, and RUNX1 mutation of AML patients in prognostic assessment, our findings confirmed for the first time that CFD could be a promising marker to predict the prognosis of AML patients. Importantly, in the present study, a nomogram to predict one-, three- and five-year survival of AML patients was successfully established which plays a significant role in predicting AML patient prognosis.

Notably, our study showed that the expression of CFD was associated with drug sensitivity of AML patients; cytarabine, doxorubicin, and bortezomib were more sensitive to AML patients with low expression of CFD, while sorafenib, lenalidomide, and bexarotene were more sensitive to AML patients with high expression of CFD, indicating that CFD could not only be used for prognostic assessment but also to guide clinical treatment.

Moreover, our findings revealed that CFD primarily correlates with monocyte infiltration in AML. Monocytes, precursors of macrophages and dendritic cells, play a crucial role in the tumor microenvironment by inducing immunotolerance, angiogenesis, and tumor cell proliferation [28]. Furthermore, monocytes can activate antigen-presenting cells and generate anti-tumor effectors that stimulate an immune response [29,30], and impaired monocyte functions may result in immunosuppression and ultimately recurrence of AML [28]. These studies suggested that high expression of CFD might promote monocyte infiltration, leading to a favorable prognosis in patients with AML.

However, there are some limitations in the current study. The clinical significance and role of CFD in AML were analyzed from the data collected in GEO, CCLE database, HPA database, LinkedOmics database, Kaplan-Meier plotter, TIMER, and CAMOIP databases. We did not verify the clinical significance and function of CFD in AML through in vitro and in vivo experiments. Therefore, the clinical significance and potential function of CFD in AML require further exploration through such experiments.

## Conclusions

Our comprehensive investigation reveals that CFD plays a significant role in AML. The elevated expression of CFD in AML cells, as compared to other tumor cell lines and normal groups, underscores its potential as a specific biomarker for AML. Importantly, higher CFD expression correlates with better OS and lower TMB in AML patients, suggesting its prognostic value. The construction of a nomogram model based on CFD expression provides a practical tool for predicting patient outcomes. Additionally, the association of CFD with drug sensitivity and monocyte infiltration highlights its potential in guiding clinical treatment strategies. Therefore, CFD stands out as not only a valuable OS prognostic biomarker but also a promising target for therapeutic interventions in AML.
